# An Unusual Presentation of Pyelonephritis: Is It COVID-19 Related?

**DOI:** 10.1007/s42399-021-00909-0

**Published:** 2021-04-24

**Authors:** L. J. van ‘t Hof, L. Pellikaan, D. Soonawala, H. Roshani

**Affiliations:** 1 Department of Urology, HagaZiekenhuis, The Hague, The Netherlands; 2 Department of Internal Medicine/Nephrology, HagaZiekenhuis, The Hague, The Netherlands

**Keywords:** Pyelonephritis, SARS-CoV-2, COVID-19, Coagulopathy, Hemorrhagic infection, Ureteral obstruction

## Abstract

In severe cases of COVID-19, late complications such as coagulopathy and organ injury are increasingly described. In milder cases of the disease, the exact time frame and causal path of late-onset complications have not yet been determined. Although direct and indirect renal injury by SARS-CoV-2 has been confirmed, hemorrhagic renal infection or coagulative problems in the urinary tract have not yet been described. This case report describes a 35-year-old female without relevant medical history who, five days after having recovered from infection with SARS-CoV-2, had an unusual course of acute pyelonephritis of the right kidney and persistent fever under targeted antibiotic treatment. A hemorrhagic ureteral obstruction and severe swollen renal parenchyma preceded the onset of fever and was related to the developing pyelonephritis. Sudden thrombotic venous occlusion in the right eye appeared during admission. Symmetrical paresthesia in the limbs in combination with severe lower back pain and gastro-intestinal complaints also occurred and remained unexplained despite thorough investigation. We present the unusual combination of culture-confirmed bacterial hemorrhagic pyelonephritis with a blood clot in the proximal right ureter, complicated by retinal vein thrombosis, in a patient who had recovered from SARS-CoV-2-infection five days before presentation. The case is suspect of a COVID-19-related etiology.

## Introduction

The outbreak of severe acute respiratory syndrome coronavirus 2 (SARS-CoV-2), recognized as a pandemic by the WHO in March 2020, has presented increasing challenges for healthcare professionals and societies. Although the understanding of the associated coronavirus disease 19 (COVID-19) is rapidly evolving, the clinical manifestation and consequences remain to be fully delineated.

While respiratory symptoms are the most apparent feature of the disease, endothelial dysfunction, abnormal coagulation parameters, and consecutive thrombosis are increasingly recognized sequelae [1, 2]. Complications that stem from such vascular aberrations occur in up to 31% of ICU patients with COVID-19 [2]. Acute kidney injury is also a common characteristic of hospitalized patients with severe COVID-19 infection [1, 3]. Although such renal injury through systemic illness, sepsis, vascular occlusion, and even direct viral damage of the nephron are increasingly documented, pyelonephritis and ureteral complications have not yet been reported in relation to a SARS-CoV-2 infection [3–5].

We present a case of a 35-year-old female without comorbidity, who, five days after recovering from COVID-19, had an unusual course of acute bacterial pyelonephritis, hemorrhagic ureteral obstruction, and persistent fever despite antibiotic treatment.

## Case Presentation

A 35-year-old female presented to the emergency room with acute constant right-sided flank pain, restlessness, vomiting, and macroscopic hematuria with blood clots for three days. Two weeks earlier, she had been diagnosed with RT-PCR-confirmed COVID-19, following a transient fever and loss of smell and taste, which had lasted until five days before presentation. At presentation, the patient did not have a raised temperature or any other symptoms. Her medical history was unremarkable except for two episodes of pyelonephritis (side unknown), one during pregnancy, of which she clearly recognized the pain. She did not use any medication.

**Fig. 1:**
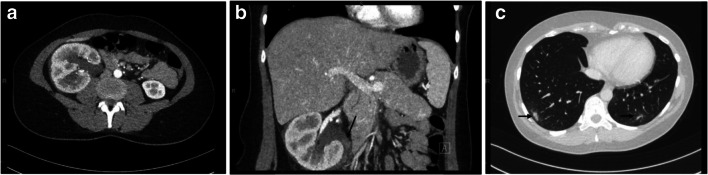
Contrast-enhanced CT scan at presentation, 100 seconds after intravenous contrast. **a**, **b** Microcalcifications and severe hydronephrosis of the right kidney. A dense mass of 75 HU, corresponding to the density of a blood clot, can be seen at the transition of the pyelum to the proximal ureter and is indicated by an arrow. A slight layer of fluid around the kidney can be seen and is suggestive of inflammation. There is a delayed arrival of contrast to the right kidney compared with the left side. In a later phase of the scan, retention of contrast in the right pyelum with delayed drainage was observed. **c** Ground glass abnormalities (arrows) in the peripheral basal lung areas which were deemed to be remnants of COVID-19

Vital functions and physical examination showed no abnormalities. Blood tests revealed a serum creatinine level of 65 μmol/L, with an eGFR (MDRD) of 90 ml/min/1.73 m^2^. Leucocytes were elevated (13.5×10^9^/L, Table 1). Urine was positive for erythrocytes, leukocytes, and nitrites. Ultrasound showed hydronephrosis of the right kidney. Contrast-enhanced computed tomography (CT) revealed an obstructive dense mass of 75HU, corresponding with a blood clot, in the proximal right ureter close to the renal pelvis. The right kidney showed severe parenchymal thickening, multiple small calcifications deep in the calyces, and extensive hydronephrosis (Fig. 1). In addition, ground-glass opacities were observed in the peripheral basal lung areas; suspect for remnants of the SARS-CoV-2 infection.

**Table 1:** Lab parameters

Test	Range	At first presentation	At start of fever	5 days before discharge
Urea, mmol/L	2.5-7.5	3.7	6.1	
Creatinine, μmol/L	50-95	65	**113**	57
estimated GFR (CKD-EPI), ml/min/1.73m^2	>83	106	**55**	116
Calcium, mmol/L	2.15-2.55	2.30		
Sodium, mmol/L	135-145	**134**		**134**
Potassium (plasma), mmol/L	3.2-4.7	3.7	4.0	4.1
Chloride, mmol/L	97-107	102		
ASAT, U/L	<30	23	25	
ALAT, U/L	<34	9	19	
LD, U/L	<248	183	**254**	
GGT, U/L	<40	11	12	
Bilirubin, total, μmol/L	5-19	9	7	
Alkaline phosphatase, U/L	40-120	66	90	
Total protein, g/L	63-83	75		
Albumin, g/L	32-48	42		
CK, U/L	10-145	48		
NT-proBNP (ng/l)	<125	93		
Hemoglobin (mmol/L)	7.2-9.5	8.6	**6.4**	
Hematocrit (L/L)	0.36-0.47	0.42	**0.32**	
Erythrocytes (×10^12^/L)	4.0-5.3	5.1	**3.8**	
MCV (fL)	83-100	83	**82**	
Trombocytes (×10^9^/L)	150-400	322	**119**	
Leucocytes (×10^9^/L)	4.0-10.0	**13.5**	**13.9**	
CRP (mg/L)	<0.5	3	**246**	**27**
Glucose (mmol/L)	3.5-9.0	5.6		
Protrombin time (sec)	9-12	10.1		
APTT (sec)	24-33	29		
D-dimer (mg/L)	<0.5	0.49		
Ferritin (μg/L)	10-100	92

The patient was admitted to the hospital with oral ciprofloxacin, adequate analgesic, ondansetron, and nadroparin prophylaxis. A urine culture was collected.

Macroscopic hematuria with clots continued intermittently on the second day of admission and lab parameters showed an increased CRP to 130 mg/L, worsening of the eGFR to 42 ml/min/1.73m^2^ and doubling of serum creatinine to 125 μmol/L (Table 1). Therefore, a nephrostomy catheter was introduced and hydration was increased.

Although the renal parameters gradually improved, the patient developed spiking fever. Antibiotic treatment was switched to intravenous cefuroxime with a one-off dose of tobramycin. A thoracic X-ray ruled out a hospital-acquired pneumonia and showed minimal basal atelectasis without any other aberrations. The urine culture showed E. coli, susceptible to ciprofloxacin and cefuroxime among others.

**Fig. 2:**
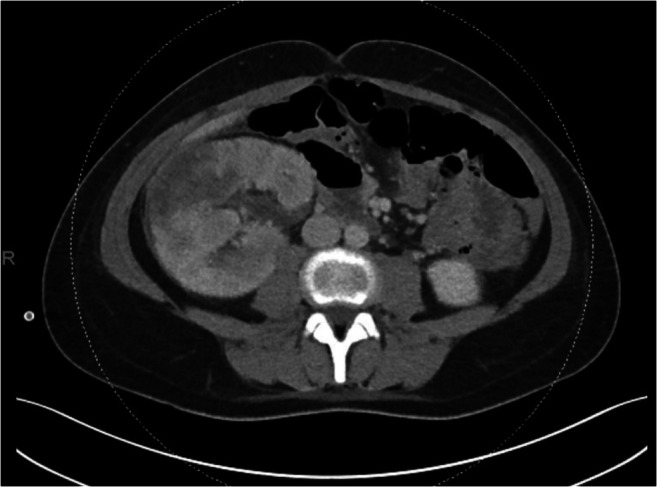
CT scan without contrast, right after the patient had developed fever. (1) Decreased volume of pyelum/hydronephrosis compared to the previous CT scan. (2) Increased area of lower density in the renal parenchyma, suspect for extension of pyelonephritis as tissue becomes increasingly hydrous. (3) Resorption of the blood clot in the proximal ureter. There are no signs of an encapsulated abscess

The spiking fever persisted and the patient developed slight anemia (hemoglobin 6.4 mmol/L). A second abdominal CT on day three showed signs of extending pyelonephritis and resorption of the ureteral blood clod, but no indication of abscesses (Fig. 2). Antibiotic treatment was switched to intravenous ceftriaxone, without subsequent improvement of fever or relief of symptoms. Nevertheless, the renal parameters gradually improved and urine production was stable and sufficient.

**Fig. 3:**
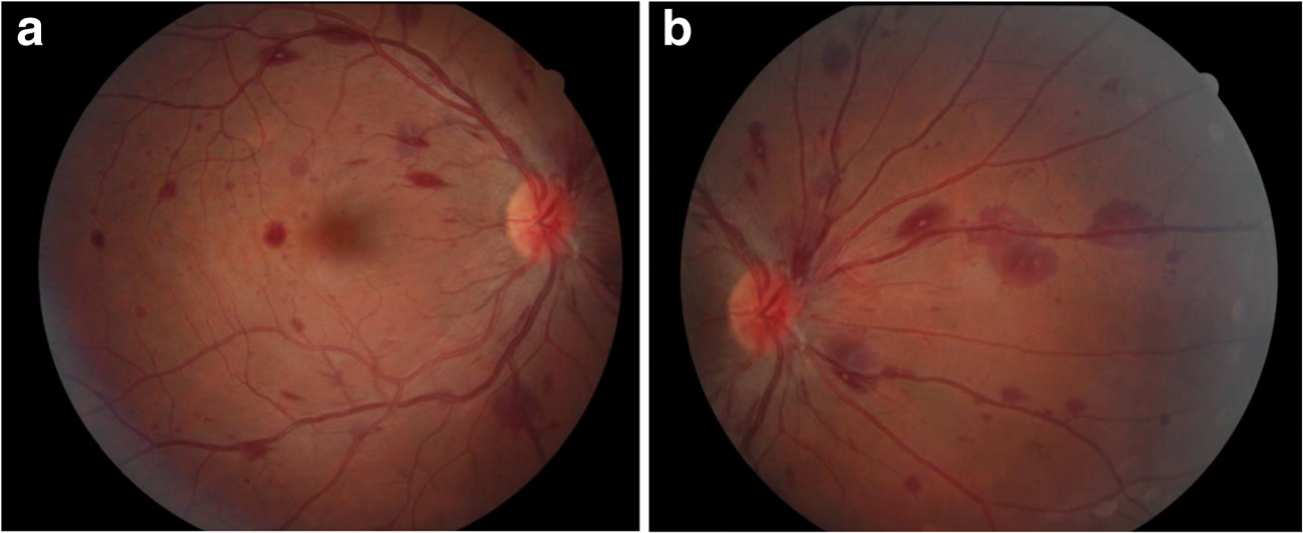
Fundoscopy of the right eye, showing bleeding across the whole retina and segmental signs of an unsharp (swollen) optic disc. **a** Right eye: central retina, macula in the center. **b** Right eye: nasal retina

Furthermore, the patient noted a decrease in vision of the right eye at day four. Fundoscopy showed diffuse retinal capillary micro-bleeding in the right eye, characteristic of a thrombotic venous occlusion (Fig. 3). There were no tell-tale signs of endophthalmitis, such as pain or anterior chamber inflammation. Beta-blocker eye droplets were prescribed. The prophylactic dosage of nadroparin was continued after consulting the vascular specialists. Follow-up through fundoscopy every other day showed no further changes during admission.

On day six, a third abdominal CT was performed and was compatible with an ongoing pyelonephritis. There were multiple hypodense areas. As a renal abscess could not be ruled out, an attempt to drain the presumed abnormality was performed. However, the procedure revealed solely swollen hydrous kidney parenchyma without purulence.

As fever spikes up to 40,4 degrees Celsius lingered through admission, another real-time reverse transcription PCR for SARS-CoV-2 from a throat swab sample was performed on day seven, with a negative result.

Due to diarrhea with mucus admixture, PCRs for Campylobacter coli/jejuni, Salmonella, Shigella, and Yersinia enterocolitica were performed, all with a negative result. The diarrhea ceased spontaneously within five days.

Sudden and recurrent symmetrical paresthesia and severe lower back pain during consecutive nights were indications to perform a cerebral and spinal MRI on day eight, which did not show any abnormalities. In addition, also in respect to the ocular vascular occlusion, a carotid and vertebral artery duplex analysis was performed on day eight, without any aberrations.

Although antibiotic treatment for sepsis was adequate, the clinical condition of the patient did not improve thus far. A multidisciplinary meeting with the departments of urology, infectious disease, microbiology, neurology, and ophthalmology did not result in a new diagnosis or full explanation of the woman's symptoms. Analysis of the persistent fever was continued with extensive analysis for systemic and (auto-)immunogenic disorders (Table 2), but none were found. The International Normalized Ratio (1.1) and the activated partial thromboplastin time (25, range 24-33 seconds) were not aberrant. The anemia remained stable throughout admission, with a minimum hemoglobin level of 6.0 mmol/L. All blood, urine, and drainage fluid culture analyses resulted in the growth of E. coli, susceptible to ciprofloxacin, cefuroxime, and ceftriaxone.

**Table 2:** Systemic and (auto)immunogenic parameters

Test	Reference range	Result
IgG4, g/l	0.08-1.4	0.34
ANCA anti-PR3	0.0-3.0	0.3
ANCA anti-MPO	0.0-5.0	0.2
ANA	0.0-1.5	< 0.5
Anti-dsDNA, U/ml	0.0-15.0	0.7
Cardiolipin IgM, U/ml	0-40	9.4
Cardiolipin IgG, U/ml	0-40	30.0
Beta-2-glycoprotein I IgM, U/ml	0-10	<0.01
Beta-2-glycoprotein I IgG, U/ml	0-10	1.3
lupus anticoagulant		Undetectable
CH-50, %	68-133	122
C1q (subfactor C1), IE/ml	81-128	129
C3 (determined with anti-C3c), g/L	0.9-1.8	1.54
C4, mg/L	150-400	180
DAT IgG	Neg	Neg
DAT C3b/3d	Neg	Neg
DAT Titer		1:1
JAN2 V617F mutation		Undetectable

The symptoms of fever and flank pain slowly decreased on the 10^th^ day of admission and the catheter was removed. The fever disappeared two days later. The patient recuperated and was in reasonable health at discharge on day 17 with oral ciprofloxacin treatment for another 10 days.

### Follow-Up

The patient returned to the ER one week later due to complaints caused by the nephrostomy catheter and was advised to hydrate and continue analgesics. There were no indications for a (recurrent) infection.

Three weeks after discharge, an antegrade pyelography revealed effective and easy passage of fluid to the bladder. Two months later, a micturating cystourethrogram (MCUG) did not show any signs of urinary reflux or anatomical deviations. Thereafter, the nephrostomy catheter was successfully removed.

**Fig. 4:**
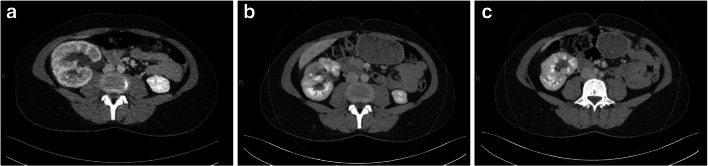
Contrast-enhanced CT scan. **a** At presentation. **b**, **c** At 4 months after discharge. There is an evident decrease in the volume of the right kidney compared to (**a**). Furthermore, notice the irregular aspect and decreased density of the surface of the interpolar region (**b**) and lower pole (**c**) at the site of the previous infection. This indicates scarring of the renal cortex. Multiple small concrements in the right kidney remain

Four months after discharge, a follow-up CT scan showed a clear decrease in the volume of the right kidney compared to the CT at admission (Fig. 4). At the site of the preceding pyelonephritis, the surface of the interpolar region and inferior pole was irregular and had a decreased density, which suggests scarring of the renal cortex. The multiple small concrements were unchanged. In one year, the patient will be seen to check her blood pressure, eGFR, and urinal protein level.

Follow-up of the eye through repetitive fundoscopy showed gradual improvement of the quantity and size of the retinal bleeding sites. However, remnants of bleeding were still visible in the peripheral retina, three months after discharge.

## Discussion

We describe the case of a young female, who, five days after recovering from COVID-19, had an unusual course of acute bacterial pyelonephritis combined with hemorrhagic ureteral obstruction and a thrombotic venous occlusion of the right retinal vein. To our knowledge, this is the first description of a possible association between hemorrhagic ureteral obstruction with pyelonephritis and COVID-19.

The exact origin of the ureteral blood clot remains unknown. The urinary tract infection may have caused hemorrhage with hematuria and consecutive formation of the obstructive blood clot in the upper urinary tract. However, the reverse, a ureteral blood clot that led to pyelonephritis, seems probable. Although hemorrhagic cystitis can be initiated by both bacteria and viruses [6], hemorrhagic pyelonephritis is extremely rare and its prevalence is unknown. Also, only 2% of all cases of spontaneous renal hemorrhage are caused by an infection [7]. Therefore, we hypothesize that the sequence of events in this patient was a hemorrhagic event or infection of the urothelium, which caused an obstructive blood clot in the ureter with subsequent urinary stasis, hydronephrosis, and bacterial infiltration of the renal parenchyma. Subsequent development of fever and acute decline in GFR is in line with this hypothesis, as is the urinary culture.

A close relation in time between the COVID-19 symptoms and the unusual hemorrhagic pyelonephritis may indicate that infection with SARS-CoV-2 of the kidney and urothelium with ensuing inflammation could be responsible for the hemorrhage in the urinary tract. Interestingly, tubular cells of the kidney and urothelial cells of the bladder express the receptor angiotensin-converting enzyme II (ACE2); the receptor utilized by the SARS-CoV-2 virus to enter human cells [8]. The kidney and urinary tract are hence considered at high risk of direct viral injury. Indeed, direct and indirect renal injury associated with SARS-CoV-2 has been confirmed [9, 10]. Similar to our described patient, 27% of patients with COVID-19 have hematuria at the time of admission, and 44% eventually develop hematuria and proteinuria during admission [3]. The presumed etiology of renal injury includes acute tubular necrosis and papillary necrosis due to a combination of hypoperfusion, cytokine release syndrome, organ crosstalk, systemic effects, and direct viral invasion [5]. Thus, renal injury, such as papillary necrosis due to COVID-19, may have provoked hematuria with ureteral clot formation.

Imaging has an important role in the diagnosis of pyelonephritis, especially if the presentation is atypical or if the response to treatment is poor [11]. At the time of presentation, there was a discrepancy between the lack of systemic symptoms, such as fever, and the morphometry of the diseased kidney on the CT scan. Although fever may be absent in the early stage of pyelonephritis, it is surprising that such symptoms were absent despite parenchymal thickening and hydronephrosis [12, 13]. The aspect of the kidney was comparable to cases of xanthogranulomatous pyelonephritis [13], in which dilated calyces lined with necrotic xanthomatous tissue extending into the renal parenchyma are seen and this cannot be ruled out in our patient. Nevertheless, the unusual course of disease suggests that the ureteral blood clot and renal injury preceded the infectious pyelonephritis.

Considering that the pathogen was susceptible to the antimicrobial treatment, the delayed clinical response was probably due to inflammation and bacterial infiltration of the renal parenchyma with impaired drainage of urine [14]. An abscess was, howbeit, ruled out by CT combined with an image-guided puncture [15]. Still, the swollen parenchyma is a “fluid reservoir”, suggesting parallels regarding subsequent intermittent fever and decreased drug penetration [14]. The recent event of COVID-19 may have contributed to the inflammation. Ronco et al. suggested that COVID-19-related renal tissue injury is based on excessive cytokine release and intrarenal inflammation [5]. Such cytokine release syndrome may lead to systemic hypotension and renal hypoperfusion, decreasing the glomerular filtration rate. In addition, an excessively high concentration of anti-inflammatory mediators may predispose the COVID-19 patient to a state of relative immunosuppression [5]. Thus, in our patient, such inflammation may have made her more prone to develop a severe bacterial pyelonephritis and may have decreased the effectiveness of the antibiotic treatment.

The follow-up, with two previous events of pyelonephritis in mind, was mainly focused on ruling out any anatomical, mechanical, or functional underlying pathophysiology. The antegrade pyelography showed effective passage of contrast and the MCUG did not show any signs of urinary reflux or anatomical deviation. The CT scan at 4 months after discharge showed decreased right kidney volume and signs of diffuse renal tissue scarring, without other abnormalities. Thus, there are no signs indicating vesicoureteral reflux or any other physical mechanisms to explain the protracted course of disease or recurrent pyelonephritis.

In COVID-19, hypercoagulation including ocular vascular occlusion is a well-described complication [1, 2, 16]. Therefore, the thrombotic retinal venous occlusion was probably related to COVID-19. No abnormalities in hemostatic parameters were found during admission and the vascular sonography of the head and neck was normal. Nevertheless, a causal relationship with the preceding COVID-19 remains plausible. Thrombotic complications, including ocular vascular occlusion, have been described while being on therapeutic anticoagulation in up to 31% of COVID-19 patients [2, 16]. Interestingly, other case reports also describe retinal vein occlusion shortly after having recovered from a COVID-19 infection [17, 18]. In addition, a case report of a COVID-19 related renal infarction suggests that a hypercoagulative state persists long after the resolution of COVID-19 [19].

In conclusion, we present the case of a 35-year-old female without comorbidity, who, five days after recovering from COVID-19, had an unusual course of acute bacterial pyelonephritis, hemorrhagic ureteral obstruction, retinal vein thrombosis, and persistent fever, despite adequate antibiotic treatment. We suggest that COVID-19-related hypercoagulation was a determinant in the causal pathway to venous retinal occlusion and that COVID-19-related renal inflammation played a causative role in ureteral hemorrhage, the subsequent hydronephrosis, and protracted course of bacterial pyelonephritis. Awareness of delayed hemorrhagic and hypercoagulative events in post-COVID-19 patients is warranted.

## Data Availability

All the authors declare data and (visual) material in this case report has not been re-used, copied, summarized, or paraphrased and has not been published before in any sort.

## Code Availability

n.a.
